# The relative importance of uncertain parameters and structural formulation for electricity systems planning in Kenya and Benin

**DOI:** 10.1016/j.isci.2025.111792

**Published:** 2025-01-13

**Authors:** Nandi Moksnes, William Usher

**Affiliations:** 1 Department of Energy Technology, KTH Royal Institute of Technology Stockholm, Brinellv. 68, Stockholm, Sweden

**Keywords:** Energy sustainability, Energy systems, Energy Modelling, Social sciences

## Abstract

When investigating access to electricity in Sub-Saharan African countries such as Kenya and Benin, which have access rates of 71% and 42% respectively (2020), modelers are faced with limited computational resources, poor data availability, and energy systems undergoing dramatic changes in the supply, transmission and distribution, and demand sides. This paper explores the relative importance of path-dependent modeling decisions, such as spatial and temporal resolution, and parametric uncertainties, such as final energy demand, discount rate, and capital costs. We use global sensitivity analysis to find that electricity demand, discount rate, and temporal and spatial resolution significantly influence the assessed results parameters. Results differ between countries showing that the model setup must use spatial and technological parameter values appropriate for the context. The results show that especially when modeling the expansion of the network, spatial and temporal resolution should be customizable and changed, even between scenarios, depending on the research question.

## Highlights

• This paper examines the relative importance of parameters in an energy system model
• Focuses on Kenya and Benin when modeling electricity access for all
• Demand, discount rate, spatial and temporal resolution are most influential

## Graphical abstract

**Figure:**
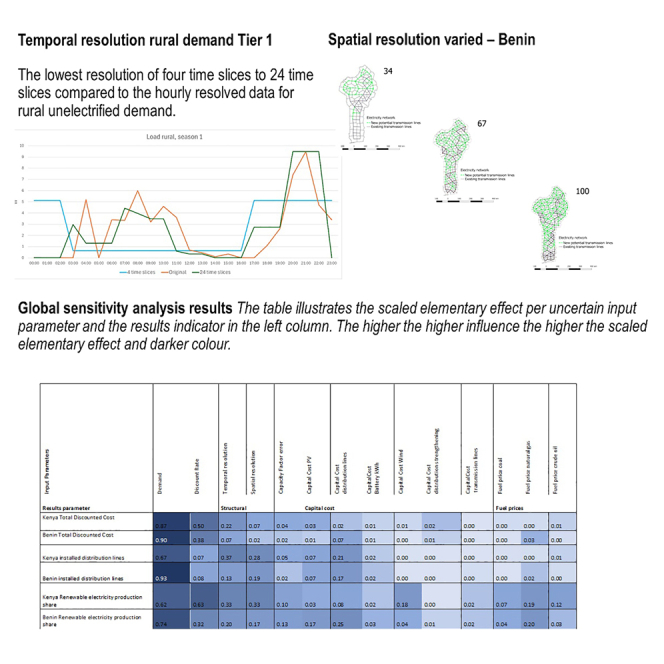

## Introduction

In response to the evolving challenges in the transition to a climate-neutral energy system, researchers in wealthy economies are developing more detailed and complex energy system optimization models (ESOMs). Using these computationally demanding models, researchers simultaneously explore the expansion and operation of mature energy systems transitioning from fossil fuels and integrating low-carbon energy sources, demand flexibility, storage and sector coupling. However, in many developing economies, while decarbonization is on the agenda, the priorities for energy system planning, the main application of ESOMs, are different. These priorities are summarized by SDG7 “providing clean energy to all”, where the latest report shows a gap of 675 million people who lack access to clean energy,^1^ almost equivalent to the population in Europe. As an energy modeling problem, energy access has specific characteristics that are different from those of mature European energy systems. For example, the transmission network has a lower extent and coverage in many sub-Saharan African countries. At the same time, the latent demand for modern energy services is unmet, while projections for energy demand increase rapidly.^2^ This raises questions about expanding transmission/distribution lines under different demands or whether distributed technologies, such as PV panels combined with batteries or mini-grids, will play an important role in reaching universal access to modern energy services by 2030.^3^

It is essential to improve the amount, quality and extent of energy system planning research for sub-Saharan Africa. Sub-Saharan Africa contains ∼560 million people^1^ who do not have access to modern energy services. At the same time, IEA reports that approx. 40 per cent of the Sub-Saharan countries have not developed official electrification plans. Due to the lack of robust electrification plans, many of the countries with the lowest electricity access rates have more difficulty accessing international concessionary financing to de-risk large investments.^1^ Most global population growth, energy demand growth and carbon emissions growth are projected to occur in sub-Saharan African countries over the next 20 years. However, there are severe limitations relating to capacity and skills, access to data and tools, and availability of scientific evidence to help inform energy system planning efforts. In this paper, we contribute insights to inform the global and African energy modeling communities.

Despite the increasing detail, models simplify reality and require researchers to consider the uncertainties and errors introduced by simplifying assumptions.^4^ Therefore, there is a need to consider the influence of these simplifying assumptions on the results of the models, both those that affect model structure and the more commonly investigated model parameters. These can be expressed as structural and parametric uncertainties – and are amenable to sensitivity analyses – to quantify the relative influence upon model results.^5^ Sensitivity analyses can identify the relative importance of parameters in a model, while uncertainty analysis captures the most probable outcome based on the uncertainty and distribution range of the input parameters.^6^ Global sensitivity analysis (GSA) can capture interactions in linear and non-linear models.^7^ ESOMs, even though they are often built upon linear programming (LP),^8^ are not linear in their inputs and results.^9^

The understanding of uncertain parameters in ESOMs and their importance in considering parametric uncertainty is largely built on case studies adopting a European context. Two models, Swiss-Energyscope^10^^,^^11^ and EnergyScopeTD^12^ are snapshot models of typical days and Calliope EU-28^13^ is a single-year ESOM. The GSA methods used in these studies are the Morris method^14^ (a one-at-the-time GSA) and Sobol’^7^ (a variance-based GSA). For multi-year ESOMs, we find UKTM,^15^ Balmorel^16^^,^^17^ and ESME UK.^18^ In these studies, the methods used were regression analysis and Morris method.^14^ The spatial resolution in ESOMs has been investigated and varied to see the impact of spatial aggregation on the overall results.^19^^,^^20^^,^^21^^,^^22^ Aggregation in temporal resolution has been studied using different methods such as typical day types or clustering methods.^23^^,^^24^^,^^25^

Simultaneous assessment of spatial and temporal resolution was assessed by Priesmann et al.^26^ who explored the accuracy and complexity of a power system optimization model (PSOM) for Germany where the spatial and temporal resolution was varied with other parameters. Priesmann et al. proposed reducing complexity in ESOMs first by grouping units (power plants and storage units), then temporal resolution and last spatial resolution. Schyska et al. investigated the relative importance of the two structural parameters temporal and spatial resolution for Europe. They found that storage need was not adequately captured with a resolution lower than 6 h. The spatial resolution was not as important as the temporal resolution in the study.^27^ Marcy et al. investigated a sub-set of spatial resolution (3, 16 and 63 regions) for the U.S. when also varying the time resolution with a clustering method and found that the root means square error increased with lower spatial resolution, particularly when clustering on hourly resolution compared to clustering day-types.^28^ Yliruka et al. assessed in a GSA one structural parameter, spatial resolution, in a heat decarbonization ESOM on a city level for the UK. The spatial resolution was compared to demand, efficiencies, technology cost and fuel prices, while the spatial resolution was found not to influence the total cost highly, it was identified as one of the most important factors for capacities of electricity, gas and heat networks.^29^ The studies above focus on high-income countries^30^ such as the UK, Norway, and Belgium.

The research gap we have identified can be summarized as follows: it needs to be clarified if the ESOM global sensitivity analysis findings based on the European context are relevant for modeling in African countries. This is due to the different structures and sizes of the respective energy systems in European and African countries, as well as the differences in the magnitude, profile, and growth of energy demands, climate/seasons, and renewable resources. While trends in techno-economic aspects such as learning rates are global in theory, developing economies experience differences in the cost of capital and other barriers (availability of skilled engineers etc.) to the adoption of capital-intensive renewable technologies. In another way, the GSA literature highlights the importance of parameter ranges in influencing GSA results. We posit that given the uncertainty ranges inhabit a different space for African countries, the GSA results will differ. Secondly, while the issue of selecting a spatial and temporal resolution can be ignored if computational resources are cheap, in many African countries, access to high-performance computing, commercial solvers is limited. As such, we imposed a computational limit on our model, which constrained the spatial and temporal resolution. Lastly, data in many African countries is often scarce or missing, so there is a need to understand the influence of model parameters and spatial and temporal resolution at which the data should be collected to prioritize data collection efforts. For example, renewable resource potential maps are often incomplete or estimated from open global datasets with limited verification.^31^^,^^32^ The limited access to computational resource and poor data quality are a phenomenon recognized in other fields as the “low-resource double bind”,^33^^,^^34^^,^^35^ so identifying where limited computational resources and data collection should be focused are valuable contributions.

Therefore, we ask the following research question: *What is the relative importance of the spatial and temporal resolution to demand, discount rate, cost of capital, capacity factor estimation error, and fuel cost parameters in an ESOM for universal access to electricity in the African context?* “African context” includes Africa-specific uncertainty ranges for key parameters, an upper bound on computational time and consequent limits on spatial and temporal resolution, and uncertainties due to data availability.

To answer this question, we use two case applications, Kenya and Benin. The two countries are representative of different modeling challenges and indicative of some of the dimensions of diversity across the continent of African. Kenya, an equatorial country in East Africa, covers an area of 585,000 km^2^. It is endowed with large, diverse, underexploited renewable energy resources, including geothermal, solar, hydropower and wind. Around 29% of the ∼52M population lacks access to electricity.^36^ The climate in Kenya is varied, with the north and northeastern regions characterized as arid desert and steppe; while the south and west of the country are temperate with two distinct seasons. Benin lies north of the equator in Western Africa. It covers an area of 115,000 km^2^. Almost 58%^36^ of the ∼11M population lacks access to electricity. The climate in the south, where the majority of the population live, is tropical savannah. The power production runs mainly on fossil fuels and imports.^37^ The topology of the high and medium voltage lines differs between the two countries. In Kenya, the electricity network is strongly concentrated in the southwest and east around large population centers such as Nairobi and Mombasa. However, the main renewable resources lie outside of the existing network in the north of the country. In Benin, the network is concentrated in the south with a transmission backbone that runs to the more sparsely populated north of the country. In both countries, there is an economic distinction between urban and rural populations and prospects for connection to existing electricity networks.

To model universal access to electricity we developed the cost-minimizing LP model generator called ‘GEOSeMOSYS’^38^ and applied it to the modeling period 2020–2040. It uses a modified version^39^ of the Open Source Energy MOdel SYStem (OSeMOSYS) model which is a dynamic capacity expansion model with programmatically variable temporal and spatial resolution for long-term energy modeling. GEOSeMOSYS simultaneously models central power plants, mini-grids, and stand-alone technology options. The framework optimises transmission and distribution line expansion to the unelectrified population in the base year and finds the optimal supply technology mix to meet demand.

**Figure 1:**
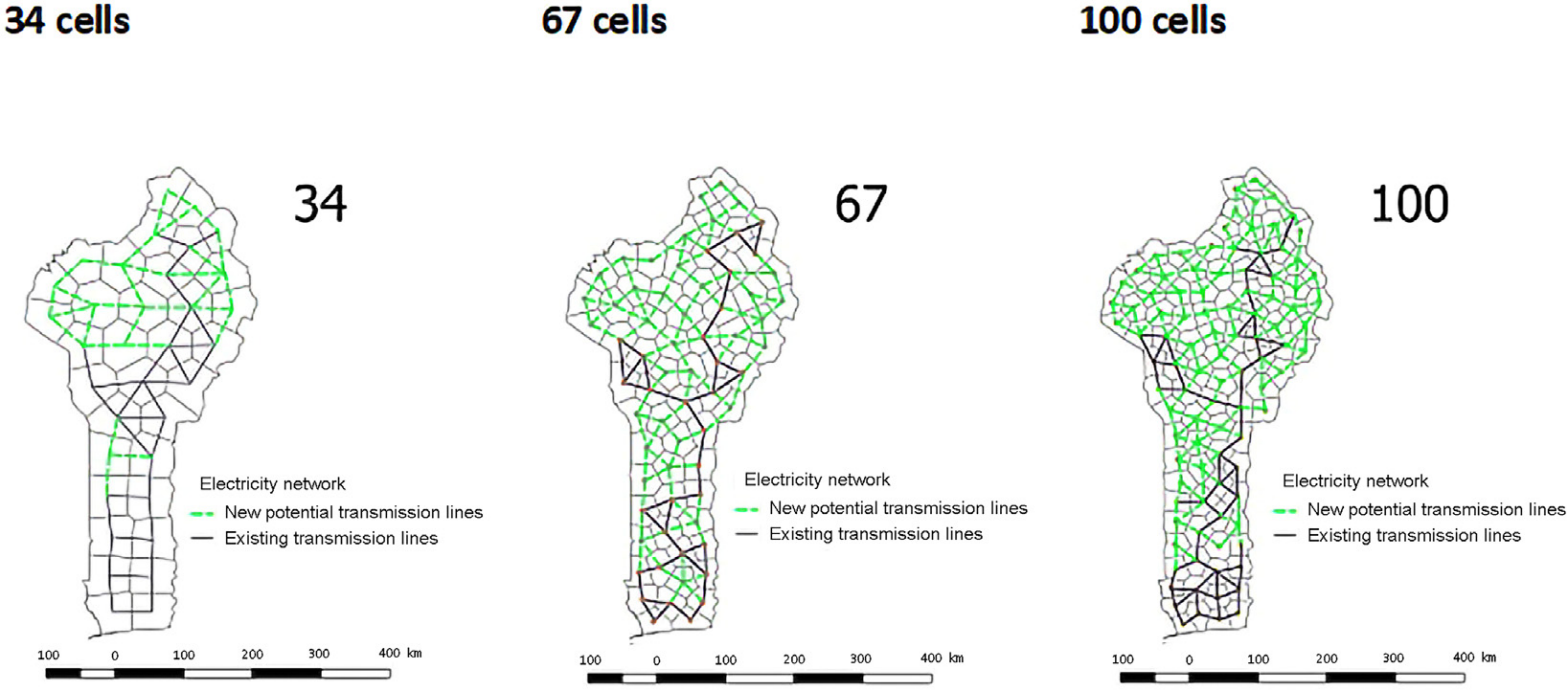
The variation of the spatial resolution for Benin with the existing transmission lines (black lines) and evaluated expansion (green lines) For more details on the electricity network see Figure S1.

For each country, we conduct a GSA in which 14 parameters vary, where the two structural parameters, spatial and temporal resolution, are assessed alongside the input parameters (see method details for details on the modeled parameters and model setup). The spatial resolution is varied between one node to the smallest cell of ∼1230 km^2^ cells for Benin and ∼5800 km^2^ cells for Kenya and includes location-specific off-grid options as well as transmission expansion from the central power plants as seen in Figure 1 for Benin.

The temporal resolution varies between 4^40^^,^^41^ and 24 annual time slices with two typical periods and 2–12 segments per day (see method details for more details on the spatial and temporal variations).

## Results

### Total discounted cost

The results in Table 1 show that for both Kenya (K) and Benin (B), the most influential parameters for total discounted cost are demand (K: $\mu_{i,SEE}^{*}$ = 0.87, B: $\mu_{i,SEE}^{*}$ = 0.90) and discount rate (K: $\mu_{i,SEE}^{*}$ = 0.50, B: $\mu_{i,SEE}^{*}$ = 0.38). For Kenya, temporal resolution (K: $\mu_{i,SEE}^{*}$ = 0.22) is ranked third most influential, while for Benin the temporal resolution (B: $\mu_{i,SEE}^{*}$ = 0.07) has the same low $\mu_{i,SEE}^{*}$ value as the cost of distribution lines. For Benin, the unscaled statistical output from the Morris method can report on the absolute actual effect ($\mu_{i}^{*}$), which amounts to 61 MUSD and 765 MUSD for Kenya when the spatial resolution change from one cell to 100 cells. The average change in these costs compared to the mean total discounted cost is 2% for Benin and 7% for Kenya. Comparing this to the literature, ranges of 1–2.75%^27^ and ∼9%^21^ and up to 23%^23^ for the total discounted cost are observed. Focusing on the temporal resolution, the absolute elementary effect ($\mu_{i}^{*}$) amounts to 278 MUSD for Benin and 2,407 MUSD for Kenya.

**Table 1:** The influence of the 14 input parameters for Kenya and Benin is measured using the scaled elementary effect ($\mu_{i,SEE}^{*}$) which is scaled by the ratio of the standard deviation of the input parameter and the standard deviation of the output results

Input Parameters	Demand	Discount Rate	Temporal resolution	Spatial resolution	Capacity Factor error	Capital Cost PV	Capital Cost distribution lines	CapitalCost Battery kWh	Capital Cost Wind	Capital Cost distribution strengthening	CapitalCost transmission lines	Fuel price coal	Fuel price natural gas	Fuel price crude oil
**Results parameter**			Structural	Structural		Capital cost	Capital cost	Capital cost	Capital cost	Capital cost	Capital cost	Fuel prices	Fuel prices	Fuel prices
Kenya Total Discounted Cost	0.87	0.50	0.22	0.07	0.04	0.03	0.02	0.01	0.01	0.02	0.00	0.00	0.00	0.01
Benin Total Discounted Cost	0.90	0.38	0.07	0.02	0.02	0.01	0.07	0.01	0.00	0.01	0.00	0.00	0.03	0.00
Kenya installed distribution lines	0.67	0.07	0.37	0.28	0.05	0.07	0.21	0.02	0.00	0.00	0.00	0.00	0.00	0.01
Benin installed distribution lines	0.93	0.08	0.13	0.19	0.02	0.07	0.17	0.02	0.00	0.00	0.00	0.00	0.02	0.00
Kenya Renewable electricity production share	0.62	0.63	0.33	0.33	0.10	0.03	0.08	0.02	0.18	0.00	0.02	0.07	0.19	0.12
Benin Renewable electricity production share	0.74	0.32	0.20	0.17	0.13	0.17	0.25	0.03	0.04	0.01	0.02	0.04	0.20	0.03

Three key result indicators are analyzed: total discounted electricity system cost, installed distance of distribution lines to unelectrified population and total renewable electricity production share.

**Table 2:** Sensitivity results from Morris method

Parameter	Description
$\mu^{*}$	Provides the mean of the absolute elementary effect.
$\mu_{i,SEE}^{*}$	Provides a scaled mean of the absolute elementary effect to allow comparison across several results parameters which has different magnitudes.
σ	Is the standard deviation which provides information if the input parameter have interaction with other parameters.

**Table 3:** Sensitivity parameters in the Morris method workflow

Type	Input parameter	Value range	Source	Unit
Structural parameter	Spatial	One node to ∼1230 km^2^ for Benin ∼5800 km^2^ for Kenya	Limited by the size of the model	Number of nodes
	Temporal resolution	4 to 24	Limited by the granularity of the hourly resolution	Clustering approach
Input parameter	Demand Unelectrified	Multi-tier framework Tier 1 to 4	^42^^,^^43^	GJ
	Discount Rate	Kenya: 8%–21% Benin: 7%–20%	^40^^,^^44^^,^^45^	Percent
	Transmission Line Cost	2 to 4	^46^	USD/kW-km
	Capital Cost distribution strengthening cost	50 to 400	Assumed value	USD/kW
	Distribution Line Cost	10,000 to 28,000	^47^	USD/km
	Capital Cost residential stand-alone PV panel	2,743 to High:1,651/Low: 671	^48^	USD/kW
	Capital Cost residential Battery (kWh)	1339 (constant) + 685 to High: 1339 (constant) + 439/Low: 1339 (constant)+ 277	^49^ with adjusted constant to Kenyan wages^50^	USD/kWh
	Capital Cost Wind power	1405 to High: 913/Low: 589	^48^	USD/kW
	Capacity Factor error PV panel and wind power	+- 3	^31^^,^^32^^,^^51^	percent
	Fuel price Natural Gas	9 to High: 8, Low: 4	^52^	USD/GJ
	Fuel price Diesel	17 to High:14, Low: 7	^52^	USD/GJ
	Fuel price Coal	5 to High: 2.9 Low: 2.1	^52^	USD/GJ

### Distance of installed distribution lines

The optimal number of kilometers of installed distribution lines to the unelectrified population in the base year is influenced the most by the demand (K: 0.67, B: 0.93). As demand increases, the better the economies of scale of expanding the grid compared to using distributed generation technologies. The results then diverge between Kenya and Benin, where for Kenya the next most influential parameter is temporal resolution (K: 0.37) followed by spatial resolution (K: 0.28), while for Benin it is the spatial resolution (B: 0.19) followed by the cost of distribution lines (B: 0.17).

These results highlight the interactions between spatial resolution, temporal resolution and the inherently spatial result of transmission and distribution line expansion. Increasing spatial resolution leads to more numerous contained “cells” where extending the transmission line needs to be cost optimal further away, leading to potentially fewer places where the distribution lines can be installed. This is partly explained by the more realistic (higher) construction and interconnection cost of extending the *transmission* lines to the (more numerous) adjacent cells which leads to higher costs to connect them. This cost is not captured in the one-node analysis where the transmission cost is applied per installed capacity. The distribution lines installation remains the same in the modeling of one node, covering the whole country. As temporal resolution increases, the temporal clusters of the time series are optimized together with location-specific data, better capturing the peaks in the system.

### Share of renewable electricity production

Due to Kenya’s large geothermal and hydropower resources, the results show that the share of renewable electricity production (REP) is relatively high for all modeled scenarios, ranging between 67% and 89% for the whole modeling period (2020–2037). As seen in Table 1, the most influential parameters for REP are discount rate (K: $\mu_{i,SEE}^{*}$ = 0.63, B: $\mu_{i,SEE}^{*}$ = 0.32) and demand (K: $\mu_{i,SEE}^{*}$ = 0.62, B: $\mu_{i,SEE}^{*}$ = 0.74). For Kenya, these are jointly followed by spatial ($\mu_{i,SEE}^{*}$ = 0.33) and temporal resolution ($\mu_{i,SEE}^{*}$ = 0.33). For Benin the cost for distribution lines ($\mu_{i,SEE}^{*}$ = 0.25), price for natural gas ($\mu_{i,SEE}^{*}$ = 0.20) and temporal resolution ($\mu_{i,SEE}^{*}$ = 0.20) are the third and joint fourth most influential.

As demand increases, more electricity generation capacity is required. Demand’s high influence on the share of renewable energy production reflects the cost-competitiveness of renewable technologies as all other parameters change. Conversely, high discount rates negatively influence the REP share as the capital intensity of renewable supply options is generally higher than for fossil-fueled supply options. For example, in Benin, when the discount rate is low, hydropower is more cost-competitive than fossil-fueled generation.

**Figure 2:**
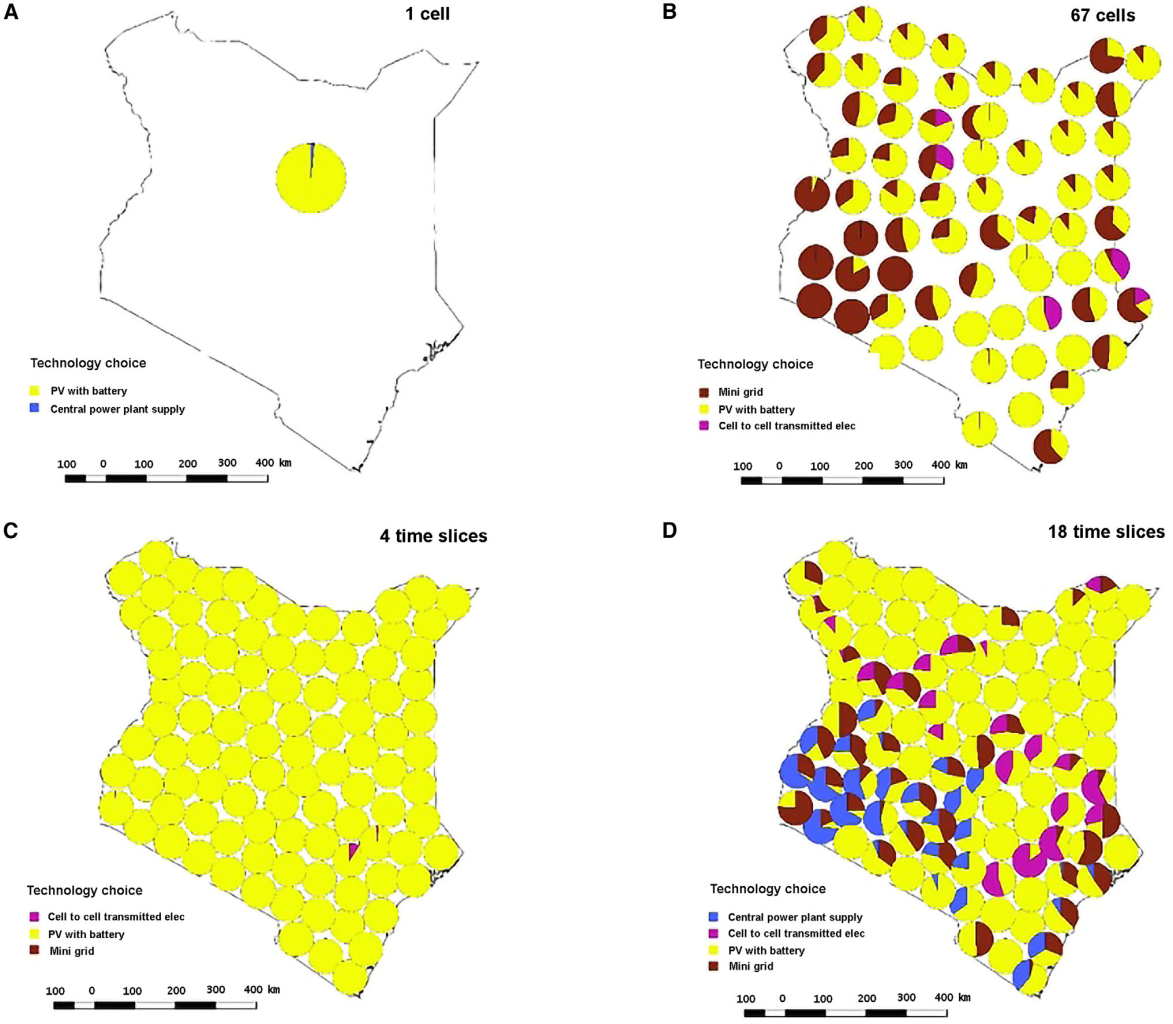
The figure illustrates the spatial and temporal resolution in the study from two perspectives for the population that lacks electricity in the base year, for the sum of the period 2020–2037 (A and B) illustrate the change in technology choice when changing the spatial resolution from 1 to 67 cells when modeling with 10 time slices, ceteris paribus. (C and D) illustrate for Kenya the results for 4 time slice to 18 time slice model for 100 cells, ceteris paribus.

Figure 2 shows how viewing the results at a technology level reveals significant variations as spatial and temporal resolution changes. The most illustrative change is that when moving from low to high spatial or temporal resolution the electricity production of mini-grid supply increases. When increasing the spatial resolution, the mini-grid electricity generation share of total electricity generation increases from 0% to 5% over the period. Similarly, for the temporal resolution when modeling at 4 time slices at resolution 100 cells, the mini-grid share of total electricity generation increases from 0% to 4.5% when increasing the temporal resolution to 18 time slices. The model can choose different mini-grid technology options, where diesel generator is one option. This leads to the insight that the influence of REP share is not directly affected by the mini-grid parameters. The optimal mini-grid technology choice is a mix of wind, solar PV, and diesel generators for both of the higher resolved scenarios.

## Discussion

**Figure 3:**
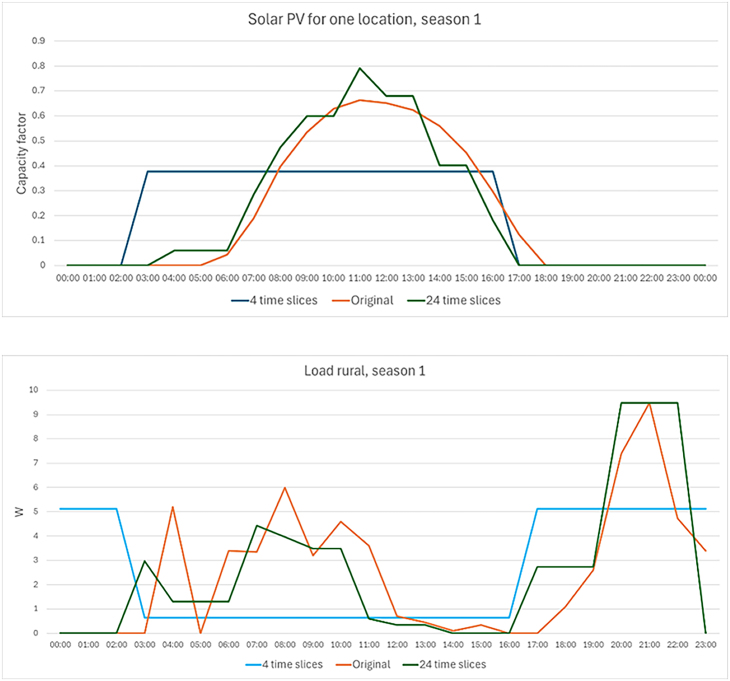
Example of time-slice representation, here for solar PV from Renewable.Ninja and the demand profile for Tier 1 level demand compared to the average hourly demand profile adapted from Narayan et al.^42^

We find that the structural parameters, temporal and spatial resolution, are influential in this study for the key results indicators. For each country and result, spatial resolution is at least as significant as the other model parameters. It has the most effect on the expansion of distribution lines and the renewable production share. The higher importance observed for temporal resolution, particularly for Kenya, can be explained by the lower resolved temporal resolution of four time slices leading to a lower peak (see Figure 3) than the higher temporal resolution. The peak is the controlling variable for the capacity requirements upon which the system’s capital cost is based. Conversely, homogeneous variation of energy potential over the studied area can motivate a lower spatially resolved model.^53^ At the same time, a lower resolved model does not capture green field expansion of the transmission lines, omitting more exact transmission and distribution costs. Furthermore, it was observed that the lower spatial and temporal resolved models also optimized less mini-grid technologies.

Schyska et al. found that the spatial resolution has an effect between the lowest (45 nodes) and highest resolution (128 nodes) of less than 4 EUR/MWh while the temporal resolution had an effect on the results of over 13 EUR/MWh when moving from hourly resolution to 12-hourly resolution for Europe.^27^ This is more than three times higher cost increase between the two analyzed parameters. Comparing the results for a fully electrified region (Europe) to the results in this study the $\mu_{i}^{*}$ for the spatial resolution was 61 MUSD for Benin and 765 MUSD for Kenya and comparing this to the temporal resolution the $\mu_{i}^{*}$ was more than three times higher for Kenya and four times higher for Benin. This can give an indication that the results are in the same order compared to Schyska et al., even though the studies differ in study design.

The spatial resolution influences the expansion of distribution lines for both countries, implying that the spatial resolution cannot be fixed to a standardized value when modeling different countries. Furthermore, due to the linearity assumption, very small amounts of transmission lines can be installed at quite a low cost. If the transmission grid would be implemented as a mixed integer linear programming, allowing only to be installed at a certain capacity, then these ‘barriers’ could potentially influence the results to a higher impact, however, this needs further investigation.

The Morris method is a computationally efficient method to understand sensitivities in a model as it does not require as many runs as other GSA methods such as Sobol’. The high sigma values observed for many of the input parameters indicate that further investigation should be conducted to identify what are the important 2^nd^- and higher-order interaction effects. Furthermore, the scaled measure of sensitivity $\mu_{i,SEE}^{*}$ for the total discounted cost shows a shift in relative importance when compared to the $\mu_{i}^{*}$ for Capital Cost distribution lines, Capital Cost PV, and Capital Cost distribution strengthening where the order is internally shifted. This highlights the limitation of the Morris method, which can only give indications of influences, especially to non-influential parameters. To reveal second and higher-order interactions, other sensitivity measures such as Soboł can be used. This, however, comes at a large computational expense as Soboł number of runs is N(*k*+2), where *N* ≈ 500–1000 and *k* are the numbers of input parameters,^7^ leading to at least 8000 scenario runs for this study. For context, running the GSA analysis for Benin and Kenya with 150 scenarios per country took ∼66 h on a 250 GB RAM computer.

The demand parameter is highly influential, and the levels of Tier 1 to Tier 4 for the unelectrified population in the base year is a large range. Tier 4 is equivalent to a household with high-power appliances, while Tier 1 is a household with some lighting and cell phone charging. The electricity demand range we consider is ambitious, but possible with adequate government support. The demand profile for the electrified demand was assumed to have the same profile for both countries, and it remained the same throughout the modeling. This is a simplification, and better data to enrich the electrified demand would give better insight into these interactions as well. As the focus of this study was to understand the pathways to universal access to electricity, the demand profiles for the unelectrified population in the base year were the focus and varied with the corresponding demand level.

The data quality also affects the results, and the transmission and distribution network dataset for Kenya is more detailed compared to Benin (see supplementary material), therefore, the open-access data used in this analysis can also influence the results when calculating the need for new distribution lines. The capacity factor error also affected the results; however, the error was implemented for the full year either up or down. It was applied to wind power as well as solar PV, with an error of 3%. A case study in Norway^51^ found that the wind farm estimation from Renewables ninja^31^ could have a much higher error margin. The discount rate was applied uniformly in the study, however, Agutu et al.^44^ suggest that off-grid solutions have a much higher cost of capital than other technology options so applying technology-specific hurdle rates could enable a more nuanced understanding of how cost of capital could affect technology deployment.

### Conclusions

A large share of the population is still living without access to electricity in Sub-Sharan Africa (∼560 million people^1^), across 40+ nations. Electricity access is not only important by itself but has also been identified as an enabler for the other SDGs.^54^ Energy models play an important role in capturing pathways and trade-offs to achieve SDG7. To provide robust results, the models need to capture highly influential parameters, where structural parameters, temporal and spatial resolution, drive computational effort. However, in many developing economies, access to computational resource, expensive commercial solvers and high-quality data is limited.

This paper highlights, therefore, the influence of the structural design of ESOMs on electricity access in Kenya and Benin. The results highlight that for the three indicators investigated, the most influential parameter is demand, with discount rate, temporal and spatial resolution and specific fuel and technology cost parameters next most influential. There are significant differences between the results for Kenya and Benin which reflect the differential starting point of the energy systems, progress toward access to energy and the unique combination of resources available. We find that both temporal and spatial resolution influence the results, however, the temporal resolution has a slightly higher influence than the spatial resolution in both case applications. This finding is important since both the spatial and temporal resolution are often set early in the modeling process and the subsequent processing of data depends on these. We propose that especially when modeling the expansion of the network, spatial and temporal resolution should be customizable and changed, even between scenarios, depending on the research question on hand.

It was also observed that, compared to other studies,^20^^,^^22^^,^^26^ the importance of the spatial resolution varies and therefore the technology mix (e.g., high penetration of PV), and other parameters in the model (e.g., congestion in the transmission lines, limits to the expansion of the network) could be explanations for the variance of the importance of the spatial resolution between the different studies and this paper. One important aspect to highlight is also that this study is applied to countries where the electricity network does not cover the whole area of the country, posing other challenges to the system. The optimal technology mix for mini-grid was very low for lower resolved spatial and temporal resolution models compared to higher resolution models. This shows a concrete change in results that is not captured in the lower resolved model.

Proper representation of the demand peak is important, and therefore implementations to better represent it can help mitigate the influence of temporal resolution if modeled in a lower temporal resolution, e.g., by adding a peak parameter to the ESOM to secure the overall peak of the system (leading to higher capital cost investments). The temporal resolution was modeled using a clustering approach; however, when modeling at a very low temporal resolution it is difficult to capture the peak without locking one of the time slices as a peak time slice (for a very limited hour) and then use the remainder three time slices to represent the yearly characteristics. This is a flaw that this study highlights, and modelers need to be aware of when the model setup is designed in this way.

Both the elementary effect of the temporal and spatial resolution showed a large economic effect on the total discounted cost but also changes in the share of renewable electricity production. This implies that working on the lowest level of temporal resolution in this study can result in higher shares of renewable electricity production and lower costs, which when increasing the resolution is not as plausible.

In conclusion, the results highlight that for the three indicators investigated—total discounted cost, distribution line length, and total renewable electricity share—the most influential parameter is demand, with discount rate, temporal and spatial resolution, and specific fuel and technology cost parameters next most influential. The results are broadly consistent across Benin and Kenya, although specific parameter priorities differ across the countries as a function of their unique geographical, resources, and socio-economic situations. These insights indicate that model development and data collection should prioritize accurate electricity demand projections, followed by increasing temporal and spatial resolution of data and modeling. Modelers should acknowledge the high influence of discount rate, and the relatively less important but still influential role of technology and fuel costs on model results.

### Limitations of the study

Future work is required to better capture more than two seasons, which can influence the results where there are seasonal changes, e.g., hydrology, wind power, and demand. Furthermore, the spatial resolution in this study was based on making the area as even as possible. Using other clustering approaches can help to simplify without affecting the results as seen in,^19^ but this requires further research on applicability for electricity access. Therefore, including spatial clustering/aggregation approaches in the GSA would prove valuable to understanding that the implementation interacts in the intended way. Apart from the structural parameters, we can also see that demand and discount rates are highly influential and a more detailed representation of these can better help us understand the evolution of the system. This can be done by first quantifying the uncertainty surrounding the most influential variables and then exploring the consequences of this uncertainty range. In this study we use a uniform discount rate, however, a technology-specific discount rate could alter the results and thus would also be a suitable future parameter to include.

## Resource availability

### Lead contact

Further information and requests for resources and materials should be directed to and will be fulfilled by the lead contact, Nandi Moksnes (Nandi@moksnes.se).

### Materials availability

No materials were used in this study.

### Data and code availability

• The study data have been deposited in https://github.com/KTH-dESA/GSA_Spatial_temporal and are publicly available as of the date of publication at https://doi.org/10.5281/zenodo.14497389. The solar and wind profiles analyze existing, publicly available data, accessible at Renewables.ninja and the GIS-data of the electricity network analyze existing, publicly available data, accessible at https://energydata.info.
• All original code has been deposited at https://github.com/KTH-dESA/GSA_Spatial_temporaland is publicly available at https://doi.org/10.5281/zenodo.14497389 as of the date of publication.
• Any additional information required to reanalyze the data reported in this paper is available from the lead contact upon request.

## Acknowledgments

We want to thank Dr. Stefano Moret for providing clarifications on the methods of scaling the elementary effect to be able to compare different output results. In addition, we want to thank the reviewers for their valuable comments to improve the manuscript.

## Author contributions

Conceptualization: N.M. and W.U.; methodology: N.M. and W.U. software: N.M.; validation: N.M. and W.U.; formal analysis: N.M.; investigation: N.M. and W.U.; data curation: N.M.; writing – original draft: N.M.; writing – review and editing: N.M. and W.U.; supervision: W.U.; project administration: N.M.

## Declaration of interests

The authors declare no competing interests.

## STAR★Methods

### Key resources table

**Table:** 

REAGENT or RESOURCE	SOURCE	IDENTIFIER
**Software and algorithms**		

This paper, GitHub.	https://github.com/KTH-dESA/GSA_Spatial_temporal	https://doi.org/10.5281/zenodo.14497389
Data	https://github.com/KTH-dESA/GSA_Spatial_temporal	https://doi.org/10.5281/zenodo.14497389

### Method details

#### Country model data

To address the paper’s research question, we use the open-source ESOM *OSeMOSYS*.^55^ OSeMOSYS has recently been applied in a high spatial resolution model for electricity access in Kenya.^38^ Moksnes et al. also provide an open-source model generator,^39^ *GEOSeMOSYS*, which allows the spatial resolution in OSeMOSYS to be varied programmatically. GEOSeMOSYS is spatially resolved in the sense that it consists of distributed electricity supply options as well as extending the existing grid to remote locations without electricity access in a multi-year analysis including the central power plant optimisation. The model generator analyses multiple aspects such as the location of the unelectrified population in the base year, and the length of needed distribution lines to connect all unelectrified households in the base year at a higher spatial resolution (1 × 1km). The model generator then aggregates this information with other data, such as PV panel and wind power capacity factors to the selected spatial resolution. For this paper, we develop the open-source code of GEOSeMOSYS further to also include varying temporal resolution, demand, discount rate, fuel costs, capacity factor error, and capital cost to run a GSA (see Table 2). To understand how the model is further spatially disaggregated we refer the interested reader to the paper.^38^

To obtain more generalisable results, two countries with low electrification rates are modeled: Kenya (29% unelectrified population) and Benin (58% unelectrified population).^36^ The general powerplant structure and future potentials, which is a pre-requisite to the GEOSeMOSYS model generator, are extracted from TEMBA^40^ for the Benin electricity sector (excluding trade with neighboring countries) and Kenya the model from the paper.^38^

#### Morris method

The spatial and temporal resolution in ESOMs drives the computational time as the higher the resolution the larger the matrix of constraints. Therefore, the driving factor of the analysis is to understand at what level can the model be reduced without compromising the results of the model. In GSA three outputs can be generated using different GSA algorithms. *Factor prioritisation*, which describes the most influential parameters where most effort and research should be given, *factor fixing*, which identifies non-influential parameters that can be removed to simplify the model, and *factor mapping*, where certain regions of interest are identified and what input parameters that drive these outcomes.^7^

The Morris method provides a basic factor prioritisation. It performs well when the number of uncertain factors is high and the computational time is long.^56^ It is most well suited for factor fixing type of analysis. It belongs to the One-At-the-Time (OAT) type of analysis, where variations around the base point are assessed, however, it overcomes some of the shortcomings that OAT has (such as the inability to capture non-linear interactions) by changing the values of all other parameters. By applying averages to different local measures, the interactions between the input parameters can be captured.^7^

The method follows *trajectories* (j), and each trajectory (10 in this study) is calculated (*k*+1) times, where *k* represents the number of input parameters. At each step of the m-th trajectory, the change in results (elementary effect) for each input parameter (i) to the j-th results parameter (Y) is calculated. To be able to compare the different results to each other (with different orders of magnitude), scaling the elementary effects as in Sin and Gearney^57^ is applied. This means that the standard deviation of the input parameter is divided by the standard deviation of the results parameter per trajectory, to act as a scaling factor (Equations 1 and 2), for an overview, please see Table 2.

Scaled elementary effect per trajectory^10^^,^^57^

(Equation 1) $${SEE}_{ij}^{m}=\frac{{\delta Y}_{j}}{{\delta x}_{i}}\frac{\sigma_{x_{i}}}{\sigma_{Y_{j}}}$$

The absolute mean scaled elementary effect per input parameter^10^^,^^57^

(Equation 2) $$\mu_{i,SEE}^{*}=\frac{1}{r}\sum_{m=1}^{r}\left|S{EE}_{i}^{m}\right|$$

To further understand the non-linear/interactions between the parameters, the standard deviation of the elementary effect $\sigma_{i}^{2}$ and the absolute mean elementary effect $\mu_{i}^{*}$ are retrieved and divided with each other (Equations 3, 4, and 5).

The absolute elementary effect per trajectory^56^

(Equation 3) $$\mu_{i}^{*}=\frac{1}{r}\;\sum_{m=1}^{r}\left|\frac{{\delta Y}_{j}}{\Delta }\right|$$

Where Δ step is set to 2/3 in this study.

Standard deviation of the elementary effect

(Equation 4) $$\sigma_{i}^{2}=\frac{1}{r-1}\;\sum_{m=1}^{r}{\left(\frac{{\delta Y}_{j}}{\Delta }-\mu \right)}^{2}$$

Interaction nonlinearity measure

(Equation 5) $$\frac{\sigma_{i}}{\mu_{i}^{*}}>1,nonlinearity\;or\;interaction\;with\;other\;parameters$$

In this paper, we want to understand if an ESOM can be reduced in size (factor fixing) without affecting the overall results. We, therefore, use the Morris method^14^^,^^56^ using the open-source sensitivity Python library SALib.^58^ To allow for calculations on the scaled elementary effects an addition was made to the SALib library.^59^

The GSA includes the two structural parameters spatial and temporal resolution, as seen in Tables 3 and 12 uncertain parametric parameters including demand, discount rate, various costs and errors in capacity factor estimates for solar and wind. The input parameters ranges, from which the samples are generated, are based on a literature review to find min and max values to find a plausible solution space. The distribution strengthening cost was assumed, starting with a cost for a transformer,^60^ however, not each added load to the system requires new investments while others require much more than just one component to keep the system in balance. All parameters are assumed to be independent of each other in the sensitivity analysis, and therefore parameters that co-vary are only represented once in the input parameters list. The two demand parameters, demand profile and total demand, are not independent from one another and, as such, follow related trajectories. Similarly, are PV panels for residential and mini-grid, and heavy fuel oil and diesel modeled in the GSA as one uncertain parameter. The modeling period is 2020–2040 for all scenarios.

#### Selection of model outputs

The results parameters chosen for this study were three: Total discounted cost, the number of installed km of distribution lines to the unelectrified population in the base year and the renewable electricity production. The total discounted cost is interesting as this is the objective function of the model and gives an understanding of what optimal solution would be preferred. The number of installed km to the unelectrified is chosen as it indicates the economic viability of connecting unelectrified households to grid/mini-grid as opposed to rooftop PV panels. Finally, as the share of renewable electricity production also is part of the SDG7, the highly influential parameters are important to capture to reach the goals.

#### Spatial resolution - Spatial clusters

The spatial cells are divided using QGIS to form even cells, meaning equal size in terms of area, over the countries. The first step to creating even-sized areas across the country is through generating 10,000–100,000 random points within the administrative boundary, this is to have many points across the country to the create clusters around. The random points are then clustered using k-means-clustering (In QGIS version 3.18.) where all generated random points have the same value. The k-means clusters are then aggregated and the centroid is extracted from these polygons. Finally, Voronoi cells are generated from the centroid and then clipped by the administrative boundaries. For Kenya, the spatial resolution is ∼5800 km^2^ and for Benin ∼1230 km^2^.

#### Demand levels

For the demand of the electrified population, the estimated low (23 TWh in 2040) and Vision scenario (44 TWh in 2040) are used for Kenya^38^ and for Benin the two demand trajectories^40^ (low 2.8 TWh, and high 8.5 TWh in 2040) are applied. The unelectrified population in the base year follow the Multi-Tier framework of Tier 1- to Tier 4^43^ the demand profiles associated with those tiers.^42^ The multi-tier framework gives a more detailed perspective on the electrification of unelectrified households and was developed by the World Bank to move beyond a binary approach to electricity access. It is divided into five tiers where lower level of electricity access is just basic lighting or mobile phone charging and then up to more continuous appliances. As the data availability for the demand profiles is limited, and there is a typical day profile that is used in the study by.^42^ Considering the different expected appliances for the different Tiers, these are mobile phones, lighting, television, and medium-power appliances which do not include AC for up to Tier 3, for Tier 4 there could be some high-power appliances such as a cooking stove use of which is again is not seasonal.^43^ As the day and night are the same length in these two countries situated on the equator, the demand for lighting can be assumed to be non-seasonal as well as the other listed appliances. Therefore, rural electricity demand is assumed to be non-seasonal.

#### Temporal resolution

The temporal resolution is clustered using the open-source Python library *tsam*.^61^ The temporal resolution varies from 4 to 24 timesteps per year, where two typical periods days represent the seasonality and the intra-day clustering, using segmentation, is varied between 2 and 12 adjacently connected clusters where in both cases a hierarchical clustering is applied. The lower bound of 4 time slices is used, similar to other studies,^40^^,^^41^ with 2 seasons and 2 daily profiles. The open-source tool tsam is designed for higher temporal resolved clusters, however, as there are studies using lower resolutions in the range of 4–24 time slices this is assessed in this study (Figure 3). Since rooftop PV panels is one of the supply options in the model, capturing the diurnal cycle is important, and therefore two intra-day clusters were important to keep at the lowest level. Since the lowest temporal resolution was set to 4 time-slices, this led to only modeling two seasons throughout all runs, and varying the intra-day clustering.

#### Transformer and household connection data

The transformer’s cost and household connection calculations were an addition to this version of the model generator: GEOSeMOSYS. It was based on the calculation from van Ruijven et al.^62^ in a 1 × 1 km resolution for both Benin and Kenya. The estimated number of LV networks per MV line is estimated through the minimum of either the number of households or the maximum of the estimated LV line length or estimated LV capacity. In addition, one MV-line is expected to be connected to the 1 × 1km settlement, and a connection is added to each one. The sum of all estimated transformers for Kenya and Benin was then multiplied by a transformer cost of 3500 USD.^60^ In addition, a connection cost per household was added of 125 USD/connection.^63^ To not create a lump sum per larger cell, the estimated cost was divided by the estimated km of the distribution line^38^ and added to the distribution line cost which is multiplied by the number of km installed lines. For Kenya, the average cost was 5646 USD/km and for Benin 7448 USD/km.

### Quantification and statistical analysis

The quantification analysis used in the study is the Morris method^14^^,^^56^ using the open-source sensitivity Python library SALib.^58^ When generating the samples for all parameters the distribution is assumed to be uniformly distributed. The results in Table 1 were calculated using Equation 1 and is also available in the SALib library.^59^
