# Supplementary material for: The relative importance of uncertain parameters and structural formulation for electricity systems planning in Kenya and Benin
Source: iScience. 2025 Jan 13;28(2):111792. doi: 10.1016/j.isci.2025.111792 (PMC11848453; doi:10.1016/j.isci.2025.111792)
Supplement: Document S1. Figure S1 [file mmc1.pdf]

iScience, Volume *28*

Supplemental information

The relative importance of uncertain parameters and structural formulation for electricity systems planning in Kenya and Benin

Nandi Moksnes and William Usher

Supplementary material

Electricity network data

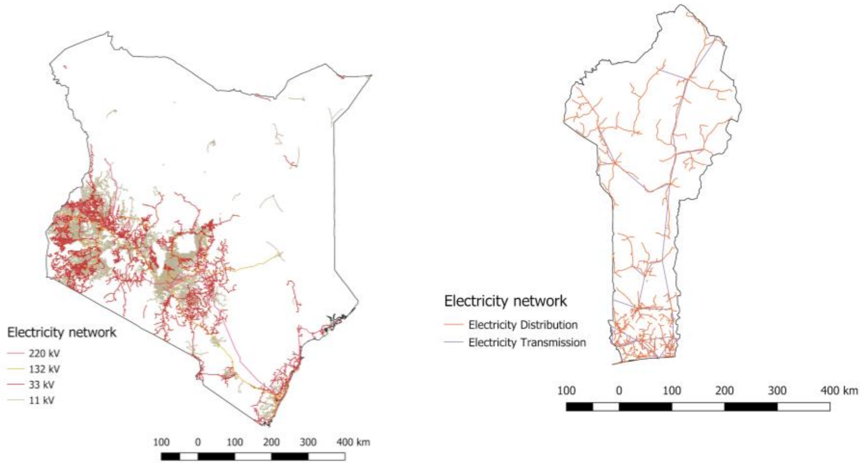

Figure S1. GIS data set for the electricity network of Kenya[1] (left) and Benin[2,3] (right) related to Figure 1.

References

1. KPLC. Kenya - Kenya Electricity Network - ENERGYDATA.INFO. 2020. Accessed June 8, 2021. https://energydata.info/dataset/kenya-kenya-electricity-network

2. Benin Electricity Transmission Network - ENERGYDATA.INFO. February 2, 2022. Accessed February 2, 2022. https://energydata.info/dataset/benin-electricity-transmission-network

3. ECOWAS Region (West Africa) — Distribution Grid - ENERGYDATA.INFO. February 2, 2022. Accessed February 2, 2022. https://energydata.info/dataset/distribution-grid-ecowas-region-west-africa
